# Correction: Liquid-liquid phase separation mediated immune evasion of respiratory syncytial virus against oligoadenylate synthetase-RNase L pathway

**DOI:** 10.1371/journal.ppat.1014193

**Published:** 2026-05-05

**Authors:** Woo Yeon Hwang, Michael G. Rosenfeld, Soohwan Oh, Young-Chan Kwon

S5 Fig was uploaded incorrectly. Please see the correct S5 Fig here.

## Supporting information

S5 Fig OAS3 and RNase L were excluded from inclusion bodies (IBs). (A) A549-RNase L KO stably expressing GFP-RNase L and mRuby2-OAS3 cells were infected with RSV A2 at an MOI of 2, stained with anti-N antibody (green) at 24 h post-infection, and imaged using an LSM 980 confocal microscope. (B) The white line indicated the rack of a line intensity profile. Scale bar, 10 μm.
(DOCX)
