# Supplementary material for: Liquid-liquid phase separation mediated immune evasion of respiratory syncytial virus against oligoadenylate synthetase-RNase L pathway
Source: PLoS Pathog. 2026 Mar 27;22(3):e1014089. doi: 10.1371/journal.ppat.1014089 (PMC13043043; doi:10.1371/journal.ppat.1014089)
Supplement: S1 Fig — (A, B) HEp-2 cells were infected with RSV A2 at an MOI of 2 and lysed at the indicated time points. rRNA cleavage and RNA integrity number (RIN) were analyzed using the RNA TapeStation System. Viral replication was examined by immunoblotting with anti-RSV N and anti-β-actin antibodies. (C, D) The A549 cells were infected with ZIKV PRVABC59 at an MOI of 2. RNA and protein were harvested and examined using the RNA TapeStation System and immunoblotting with anti-ZIKV NS3 and anti-β-actin antibodies. (DOCX) [file ppat.1014089.s001.docx]

**
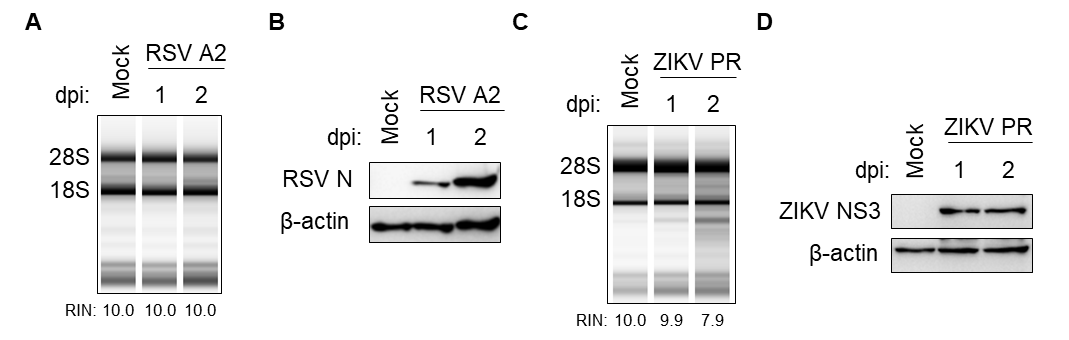
**

**S1 Fig. RSV infection fails to activate the OAS–RNase L pathway, whereas ZIKV infection triggers.** (A, B) HEp-2 cells were infected with RSV A2 at an MOI of 2 and lysed at the indicated time points. rRNA cleavage and RNA integrity number (RIN) were analyzed using the RNA TapeStation System. Viral replication was examined by immunoblotting with anti-RSV N and anti-β-actin antibodies. (C, D) The A549 cells were infected with ZIKV PRVABC59 at an MOI of 2. RNA and protein were harvested and examined using the RNA TapeStation System and immunoblotting with anti-ZIKV NS3 and anti-β-actin antibodies.
