# Supplementary material for: Liquid-liquid phase separation mediated immune evasion of respiratory syncytial virus against oligoadenylate synthetase-RNase L pathway
Source: PLoS Pathog. 2026 Mar 27;22(3):e1014089. doi: 10.1371/journal.ppat.1014089 (PMC13043043; doi:10.1371/journal.ppat.1014089)
Supplement: S2 Fig — The A549 cells were transfected with the OASs isoforms 24 h before RSV infection at an MOI of 2. At 2 d post-infection, the RNA was purified and analyzed using the RNA TapeStation System. (DOCX) [file ppat.1014089.s002.docx]

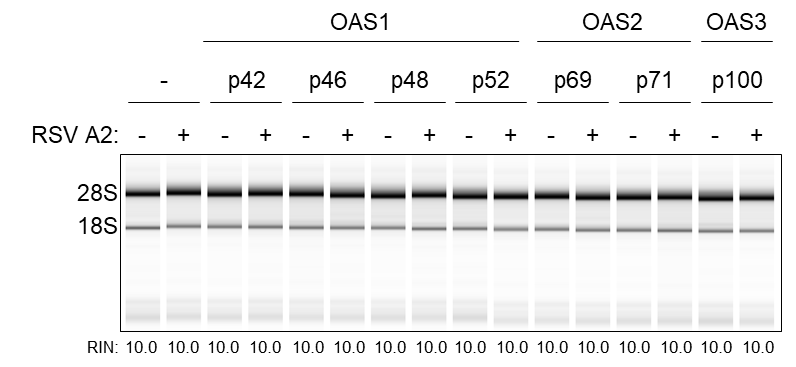


**S2 Fig. RNase L was not activated in the RSV-infected A549 cells overexpressing OAS isoforms.** The A549 cells were transfected with the OASs isoforms 24 h before RSV infection at an MOI of 2. At 2 d post-infection, the RNA was purified and analyzed using the RNA TapeStation System.
