# Supplementary material for: Liquid-liquid phase separation mediated immune evasion of respiratory syncytial virus against oligoadenylate synthetase-RNase L pathway
Source: PLoS Pathog. 2026 Mar 27;22(3):e1014089. doi: 10.1371/journal.ppat.1014089 (PMC13043043; doi:10.1371/journal.ppat.1014089)
Supplement: S3 Fig — (A) overview about detecting dsRNA through IFA and dot-blotting. (B) Total RNA from the mock-, RSV-, and ZIKV-infected cells was transferred onto nylon membranes. The dsRNA was detected using an anti-dsRNA antibody (9D5). Schematic overviews for these experiments were created with BioRender.com. Created in BioRender. Kwon, Y. (2026) https://BioRender.com/u5t1cmx. (DOCX) [file ppat.1014089.s003.docx]

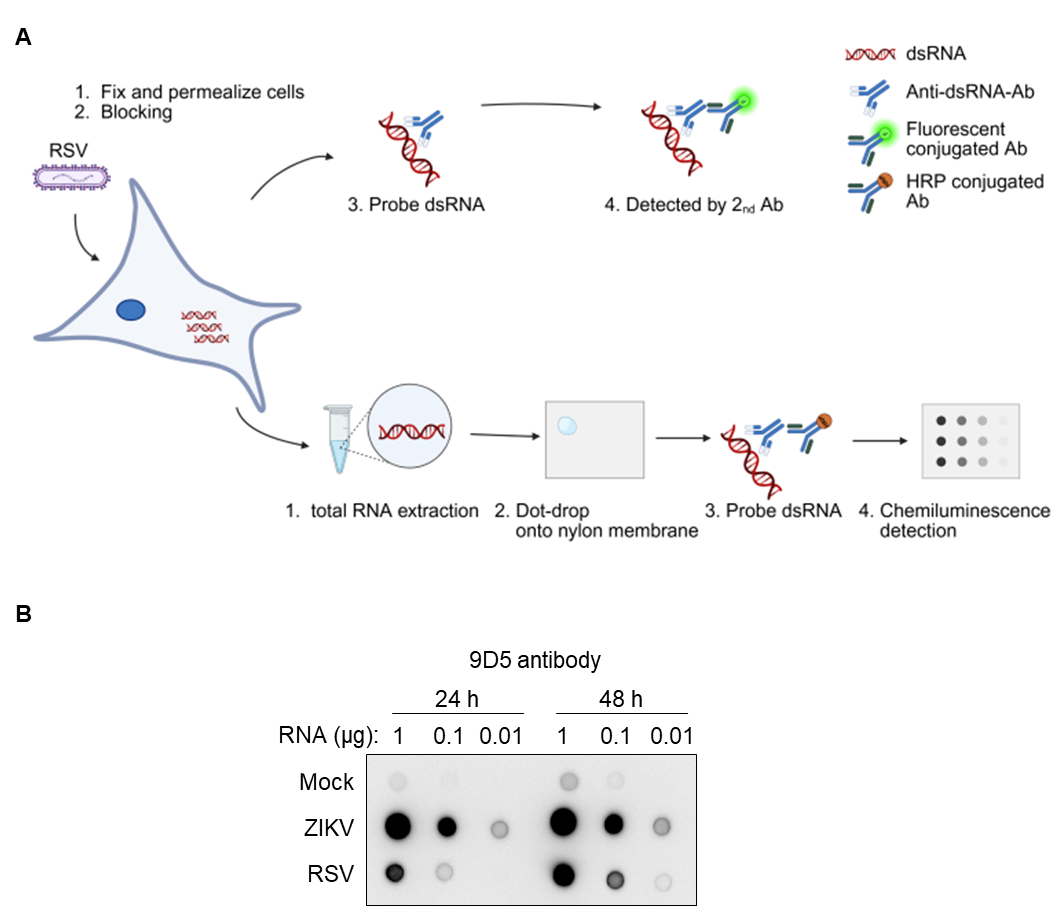


**S3 Fig. The dsRNA detection in total RNA extracted from the RSV-infected cells.** (A) overview about detecting dsRNA through IFA and dot-blotting. (B) Total RNA from the mock-, RSV-, and ZIKV-infected cells was transferred onto nylon membranes. The dsRNA was detected using an anti-dsRNA antibody (9D5). Schematic overviews for these experiments were created with BioRender.com.
