# Supplementary material for: Liquid-liquid phase separation mediated immune evasion of respiratory syncytial virus against oligoadenylate synthetase-RNase L pathway
Source: PLoS Pathog. 2026 Mar 27;22(3):e1014089. doi: 10.1371/journal.ppat.1014089 (PMC13043043; doi:10.1371/journal.ppat.1014089)
Supplement: S4 Fig — (A) A549 cells were infected with RSV A2 at an MOI of 2 and stained with anti-RSV N antibody (green) at the indicated time points. (B) A549 cells were infected with RSV A2 at an MOI of 2 and probed with anti-RSV N antibody (green), anti-RSV P (red) and anti-RSV M2-1 (red) indicated time points. Scale bar, 20 μm. (DOCX) [file ppat.1014089.s004.docx]

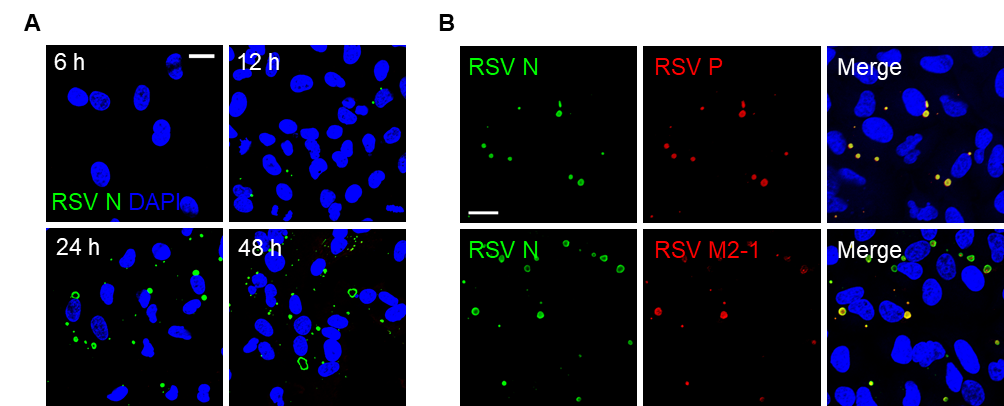


**S4 Fig. Progressive growth of RSV inclusion bodies and recruitment of N, P, and M2-1 proteins.** (A) A549 cells were infected with RSV A2 at an MOI of 2 and stained with anti-RSV N antibody (green) at the indicated time points. (B) A549 cells were infected with RSV A2 at an MOI of 2 and probed with anti-RSV N antibody (green), anti-RSV P (red) and anti-RSV M2-1 (red) indicated time points. Scale bar, 20 μm.
