# Supplementary material for: Liquid-liquid phase separation mediated immune evasion of respiratory syncytial virus against oligoadenylate synthetase-RNase L pathway
Source: PLoS Pathog. 2026 Mar 27;22(3):e1014089. doi: 10.1371/journal.ppat.1014089 (PMC13043043; doi:10.1371/journal.ppat.1014089)
Supplement: S6 Fig — The A549 cells were infected with RSV A2 at an MOI of 2. Before the cells were fixed 24 h after infection, they were treated with 5% 1,6-HD at the indicated times. Cells were stained with anti-RSV P antibody (green) and anti-dsRNA (9D5, red). Scale bar, 20 μm. (DOCX) [file ppat.1014089.s006.docx]

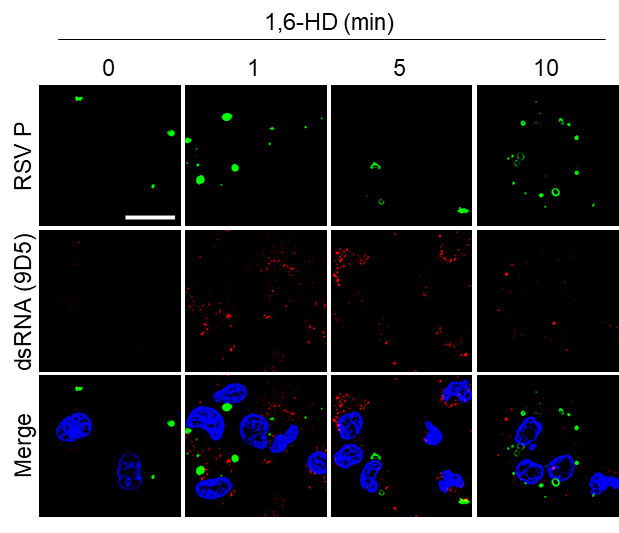


**S6 Fig. Disruption of inclusion bodies (IBs) with an LLPS inhibitor induces dsRNA leakage into the RSV-infected cells.** The A549 cells were infected with RSV A2 at an MOI of 2. Before the cells were fixed 24 h after infection, they were treated with 5% 1,6-HD at the indicated times. Cells were stained with anti-RSV P antibody (green) and anti-dsRNA (9D5, red). Scale bar, 20 μm.
