# Supplementary material for: Liquid-liquid phase separation mediated immune evasion of respiratory syncytial virus against oligoadenylate synthetase-RNase L pathway
Source: PLoS Pathog. 2026 Mar 27;22(3):e1014089. doi: 10.1371/journal.ppat.1014089 (PMC13043043; doi:10.1371/journal.ppat.1014089)
Supplement: S7 Fig — (A–B) A549 and NHBE cells were exposed to 1,6-HD or hypotonic shock, stained with anti-dsRNA antibody (red), and imaged using an LSM 980 confocal microscope. Scale bar, 10 μm. (DOCX) [file ppat.1014089.s007.docx]

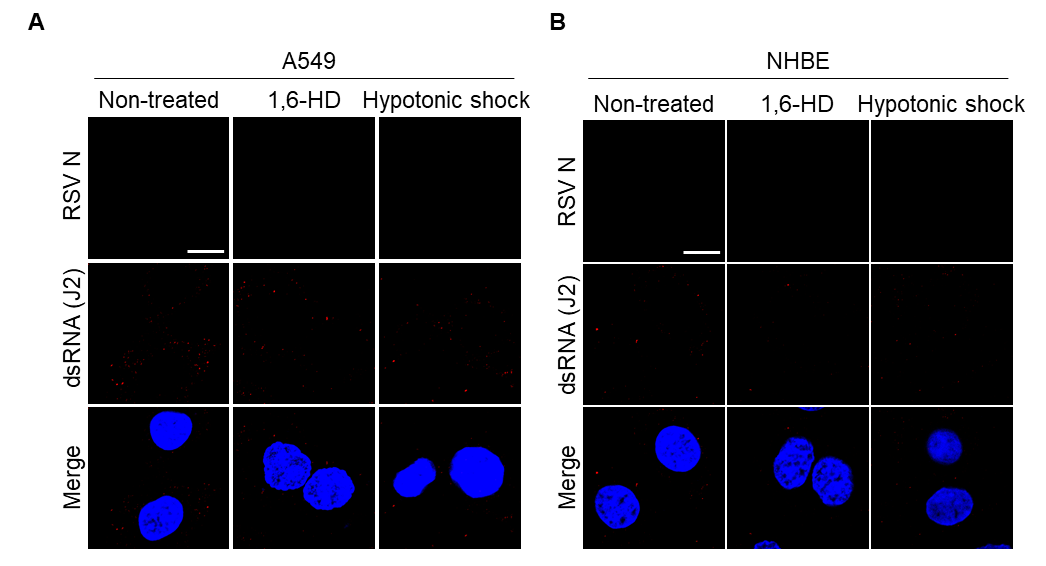


**S7 Fig. 1,6-hexanediol (1,6-HD) or hypotonic shock do not induce host dsRNA.** (A–B) A549 and NHBE cells were exposed to 1,6-HD or hypotonic shock, stained with anti-dsRNA antibody (red), and imaged using an LSM 980 confocal microscope. Scale bar, 10 μm.
