# Supplementary material for: Liquid-liquid phase separation mediated immune evasion of respiratory syncytial virus against oligoadenylate synthetase-RNase L pathway
Source: PLoS Pathog. 2026 Mar 27;22(3):e1014089. doi: 10.1371/journal.ppat.1014089 (PMC13043043; doi:10.1371/journal.ppat.1014089)
Supplement: S8 Fig — (A–B) A549 and NHBE cells were infected with RSV A2 at an MOI of 2. At 24 h post-infection, cells were exposed to 1,6-HD or hypotonic shock for 5 min, followed by a 24 h recovery period. Total RNA was then harvested and analyzed using the RNA TapeStation System. (DOCX) [file ppat.1014089.s008.docx]

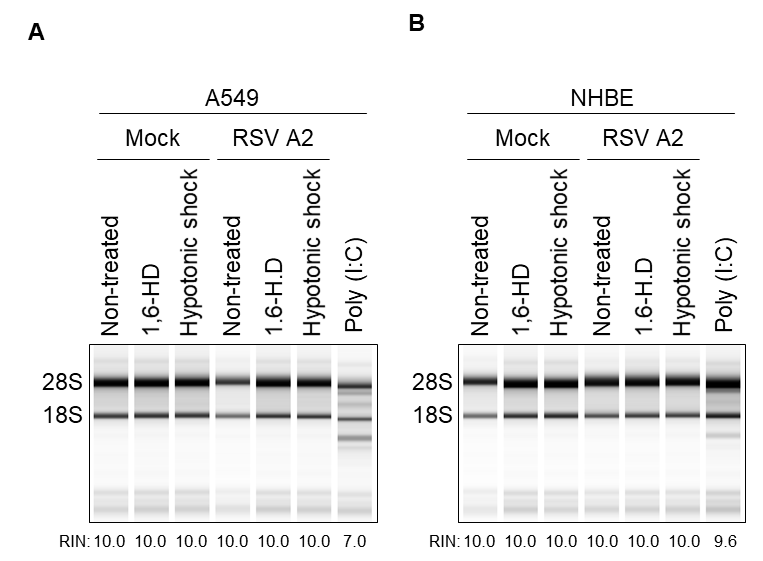


**S8 Fig. LLPS disruption does not induce rRNA cleavage during RSV infection.** (A–B) A549 and NHBE cells were infected with RSV A2 at an MOI of 2. At 24 h post-infection, cells were exposed to 1,6-HD or hypotonic shock for 5 min, followed by a 24 h recovery period. Total RNA was then harvested and analyzed using the RNA TapeStation System.
