# Supplementary material for: Liquid-liquid phase separation mediated immune evasion of respiratory syncytial virus against oligoadenylate synthetase-RNase L pathway
Source: PLoS Pathog. 2026 Mar 27;22(3):e1014089. doi: 10.1371/journal.ppat.1014089 (PMC13043043; doi:10.1371/journal.ppat.1014089)
Supplement: S9 Fig — (A) The total RNA was purified from mock- and RSV-infected cells and transfected into A549 cell with 10 μg. At 24 h after-transfection, the RNA was assessed using the RNA TapeStation System. (B) After dsRNA purification, dsRNA enrichment was confirmed by dot blotting using an anti-dsRNA antibody (9D5). (DOCX) [file ppat.1014089.s009.docx]

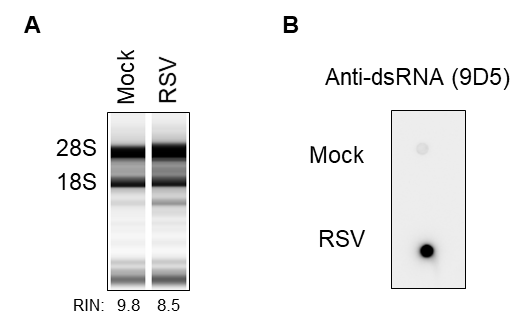


**S9 Fig. rRNA cleavage by transfection of the total RNA extracted from the RSV-infected cells and Naked dsRNA purified from the RSV-infected cells.** (A) The total RNA was purified from mock- and RSV-infected cells and transfected into A549 cell with 10 μg. At 24 h after-transfection, the RNA was assessed using the RNA TapeStation System. (B) After dsRNA purification, dsRNA enrichment was confirmed by dot blotting using an anti-dsRNA antibody (9D5).
