# Supplementary material for: Liquid-liquid phase separation mediated immune evasion of respiratory syncytial virus against oligoadenylate synthetase-RNase L pathway
Source: PLoS Pathog. 2026 Mar 27;22(3):e1014089. doi: 10.1371/journal.ppat.1014089 (PMC13043043; doi:10.1371/journal.ppat.1014089)
Supplement: S10 Fig — (A–B) A549 and NHBE cells were infected with RSV A2 at an MOI of 2. At 24 h post-infection, cells were treated with 5 μM CPM for 1 h and then exposed to 1,6-HD or hypotonic shock for 5 min, followed by staining with anti-RSV N antibody (green) and anti-dsRNA (J2, red). Scale bar, 10 μm. (DOCX) [file ppat.1014089.s010.docx]

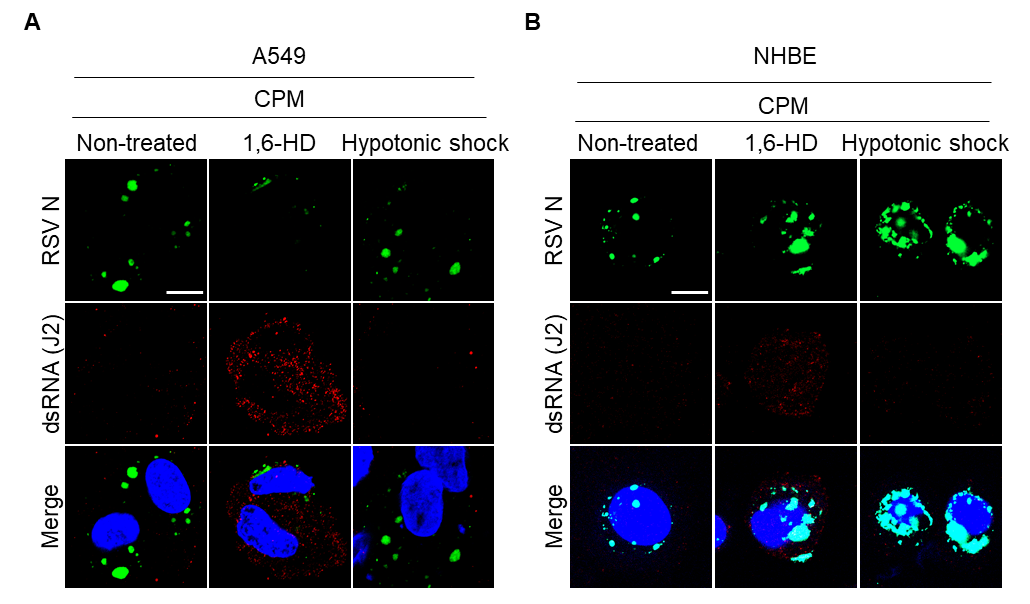


**S10 Fig. Cyclopamine (CPM) blocks hypotonic shock–induced dsRNA release but not 1,6-hexanediol (1,6-HD).** (A–B) A549 and NHBE cells were infected with RSV A2 at an MOI of 2. At 24 h post-infection, cells were treated with 5 μM CPM for 1 h and then exposed to 1,6-HD or hypotonic shock for 5 min, followed by staining with anti-RSV N antibody (green) and anti-dsRNA (J2, red). Scale bar, 10 μm.
