# Supplementary material for: Liquid-liquid phase separation mediated immune evasion of respiratory syncytial virus against oligoadenylate synthetase-RNase L pathway
Source: PLoS Pathog. 2026 Mar 27;22(3):e1014089. doi: 10.1371/journal.ppat.1014089 (PMC13043043; doi:10.1371/journal.ppat.1014089)
Supplement: S1 Table — (DOCX) [file ppat.1014089.s011.docx]

**S1 Table. qRT-PCR primers and probes.**

| **Target** | **Name** | **Sequence 5’-3’** |
| --- | --- | --- |
| RSV F | Primer 1 | AACAGATGTAAGCAGCTCCGTTATC |
|  | Primer 2 | GATTTTTATTGGATGCTGTACATTT |
|  | Probe | TGCCATAGCATGACACAATGGCTCCT |
| IFN β | Primer 1 | GCCATCAGTCACTTAAACAGC |
|  | Primer 2 | GAAACTGAAGATCTCCTAGCCT |
|  | Probe | TGAAGCAAT/Zen/TGTCCAGTCCCAGAGG |
| OAS1 | Primer 1 | GATGAGCTTGACATAGATTTGGG |
|  | Primer 2 | GGTGGAGTTCGATGTGCTG |
|  | Probe | CCTTTGATGCCCTGGGTCAGTTGA |
| OAS2 | Primer 1 | CAGATCAATGAGCCCTGCATA |
|  | Primer 2 | GAAATCCAAAGTCCTCAACGAAAG |
|  | Probe | CAGCTGACC/ZEN/CAGTGCATTAAAGGC |
| OAS3 | Primer 1 | CTGTAGCTGTGGATGAGGTC |
|  | Primer 2 | GTGTGGACTTTGATGTGCTG |
|  | Probe | TAGACTTGAGAGCTGGGCCTGGA |
| GAPDH | Primer 1 | TGTAGTTGAGGTCAATGAAGGG |
|  | Primer 2 | ACATCGCTCAGACACCATG |
|  | Probe | AAGGTCGGAGTCAACGGATTTGGTC |
