# Supplementary material for: Liquid-liquid phase separation mediated immune evasion of respiratory syncytial virus against oligoadenylate synthetase-RNase L pathway
Source: PLoS Pathog. 2026 Mar 27;22(3):e1014089. doi: 10.1371/journal.ppat.1014089 (PMC13043043; doi:10.1371/journal.ppat.1014089)
Supplement: Attachment — Submitted filename: Response to reviewers comment.docx [file ppat.1014089.s013.docx]

Dear Editor,

We would like to thank you for your consideration of our manuscript entitled "Liquid-liquid phase separation mediated immune evasion of respiratory syncytial virus against oligoadenylate synthetase-RNase L pathway" for publication in *PLOS Pathogens*. We are pleased by the interest shown by both you and the reviewers. We readily acknowledge that the reviewers' criticisms enhance the clarity and scientific rigor of our manuscript, and we thank them for their diligence. The comments are constructive and help improve the manuscript significantly. We have provided additional data, interpretations, and contextualization to address the reviewers' concerns. Below are our point-by-point responses to each comment.

Thank you for your consideration. We hope that you will concur with our final revisions and consider a positive decision, as all of the concrete concerns raised by the reviewers during the original review have now been fully addressed. In the revised manuscript, we have clarified and strengthened the central significance of our study by demonstrating how liquid–liquid phase separation of RSV inclusion bodies functions as a critical organizational mechanism that shields viral dsRNA from innate immune sensing, thereby enabling evasion of the OAS–RNase L antiviral pathway. We believe that these findings provide a clear mechanistic framework linking viral condensate biology with intracellular innate immune evasion and will be of broad interest to the readership of *PLOS Pathogens*.

Sincerely,

Young-Chan

**Reviewers' Comments:**
Reviewer's Responses to Questions

**Part I - Summary**
Please use this section to discuss strengths/weaknesses of study, novelty/significance, general execution and scholarship.

Reviewer #1:

The manuscript from Hwang et el. describes how RSV facilitates LLPS driven IBs in structurally sequestering viral RNA facilitating evasion of RNase dependent genomic RNA degradation by OAS-RNase L antiviral pathway. The study is well planned, backed up by sufficient supporting data, and clear illustration of results. Overall, the manuscript is well written, and the results are logically interpreted. The authors may please consider the following comments for minor clarification.

Response: We thank the reviewer for their thoughtful consideration and constructive comments, which have significantly improved our manuscript. We have carefully addressed each comment and incorporated the corresponding revisions into the relevant sections of the manuscript. A detailed point-by-point response to the reviewer's comments is provided below.

Specific Comments:
1. The authors need to briefly discuss physiological relevance with respect to the nature of immune evasion with respect to the outcome from respiratory immune response.

Response: We thank the reviewer for this comment, which has improved our manuscript. We have added a brief description of the immune evasion strategies employed by respiratory viruses and their mechanisms of action in the Discussion section. (lines 262-268, revised manuscript)

2. P. 5, line 4: What is meant by wild-type A549 cells? Line 13: The S2Fig needs little more clarification to match the statement from the results.

Response: Thank you for your valuable suggestions. The term “wild-type” was indeed inappropriate, and we have removed it from the manuscript (line 89, revised manuscript). In addition, we have revised the explanation of Supplementary Figure 2 (S1C and D, revised manuscript) to provide more detailed information. (line 97, S1C and D Fig, revised manuscript)

3. P. 7, 2nd paragraph: Rewarding the subheading of this paragraph will make the concluding result in line.

Response: Thank you for your helpful advice. We have revised the subheading of that paragraph to align it with the conclusion of the results. (line 135, revised manuscript)

4. P. 9, Line 11: Why did anti-dsRNA (J2 Red) not detect RSV dsRNA?

Response: Thank you for this question. The dsRNA generated during RSV replication is presumed to be sequestered within inclusion bodies (IBs), which likely reduces the accessibility of antibodies to factors located inside the IBs. In the same context, staining with anti-RSV N antibody produced a hollow droplet-like pattern (S4A and B Fig), consistent with limited antibody access to dsRNA within the interior of IBs.

5. Fig. 3, panel A and Fig. 6, panel A may appear little excess to some readers, although the schematics at a glance help to clarify the experimental approaches. The authors may add appropriate wordings in text for justification of the separate panels. Some of the supplementary figures could have been brought in context to the main result areas.

Response: We thank you for your thoughtful and insightful advice. We carefully considered your suggestion regarding the schematic graphics and thoroughly evaluated whether to move them to the supplementary section. Therefore, we decided to move Fig 3 to supplementary section (S3A Fig, revised manuscript) and retain the schematic graphics with Fig 6, as we believe they help improve the clarity of our manuscript.

6. Fig. 6, panel B and S6B: Why do the blots show hollow vesicular staining by anti-dsRNA?

Response: We thank you for your interest in this observation. This phenomenon typically occurs at low dsRNA concentrations. Notably, the hollow vesicular staining pattern was not observed in spots containing high dsRNA concentrations (Fig. 3B, S3B Fig, revised manuscript). We attribute this to a physical process known as the coffee-ring effect, which arises during droplet evaporation on membrane surfaces and is particularly pronounced at lower analyte concentrations (DOI: 10.1038/39827). In our dot blot protocol, RNA samples are applied as droplets onto a nitrocellulose membrane and allowed to air-dry. During evaporation, capillary flow drives the dsRNA-containing solution radially outward from the droplet center toward the pinned edge. This movement causes dsRNA molecules to accumulate at the periphery, resulting in the characteristic hollow or ring-shaped staining pattern. At higher dsRNA concentrations, sufficient molecules are present to occupy the entire spotted area—both center and edge—thereby producing a uniform, solid staining appearance.

7. The impact of studied immune evasion pathway in context to the other immune regulatory mechanisms may be briefly discussed.

Response: We thank you for your valuable advice. We have proposed the novel mechanism in which LLPS functions as an immune evasion strategy during viral infection. We believe that this strategy is not limited in OAS-RNase L pathway or RSV. Other pathogens may also employ LLPS to escape the immune system. We have mentioned this point in the last paragraph of the Discussion section and highlighted it accordingly. (lines 260-262, revised manuscript)

Reviewer #2: In this manuscript, Hwang and colleagues investigate how Respiratory syncytial virus (RSV) prevents the activation of the OAS-RNase L pathway. They show that RSV-infected cells have increased dsRNA by dot blot analyses but do not display RNase L activation based on the lack of RNase L-dependent of ribosomal RNA cleavage. The authors show that dsRNA is sequestered into inclusion bodies (IBs), which are susceptible to 1,6-hexanediol (1,6-HD) indicating that the IBs are liquid-liquid phase separation. Re-introduction of RNA isolated from RSV-infected cells can activate RNase L. Combined, the authors argue that RSV dsRNA replication intermediates are sequestered within IBs, and that this limits the ability of RNase L to activate.

Overall, the data support their conclusions, and the manuscript is well-written. While it is already known that many negative strand RNA viruses do not activate RNase L, and it has long been assumed that inclusion bodies prevent sensing of viral RNA substrates by pattern recognition receptors such as OAS proteins, this study formally attempts to address this, which is impactful to the field of intracellular innate immunology and virology. However, a major limitation of this study is that the authors do not provide a mechanism by which the RSV IBs prevent RNase L activation. To increase the impact of this study, the authors should consider exploring the possibility that the IBs exclude OAS and RNase L proteins, thus limiting the ability of OAS protein and RNase L to condense on viral dsRNA and activated (DOI: 10.1126/scisignal.adi9844; DOI: 10.1101/gad.352725.125). The authors should consider showing that OAS3 and RNase L are excluded from RSV IBs by fluorescent microscopy, and that the interaction between dsRNA and OAS proteins by proximity labeling assay (PLA) does not occur in IBs.

This review was written by James Burke (University of Florida Scripps Institute), and I am happy to provide any reagents that would be helpful (A549 cells that stably express GFP-RNase L, RFP-OAS3, etc).

Response: We sincerely thank the reviewer for their thoughtful evaluation and constructive comments, which have greatly improved our manuscript. We also deeply appreciate your generous suggestion in providing the reagent containing the stably expressing cell line (line 359 in M&M section). We fully agree with your comment. To examine the relationship between condensates induced by OAS3 and RNase L and RSV, we performed additional experiments.

We examined condensate formation in different cellular contexts. In cells transfected with poly(I:C), we observed robust OAS3-induced condensate formation. In contrast, RSV-infected cells did not exhibit these condensates under identical detection conditions (lines 120-127, Fig 1F, revised manuscript). OAS3 and RNase L were excluded from IBs (line 195, S5 Fig, revised manuscript). Furthermore, when we ectopically activated OAS-RNase L pathway using poly I:C in RSV‑infected cells, the OAS3‑induced condensates were still observed, but did not overlap with the IBs (line 143, Fig 2C, revised manuscript). Despite extensive optimization efforts, we were unable to reliably detect RNase L condensates under OAS-activating conditions using currently available reagents.

Minor comments:
1. Does 1,6-hexanediol (1,6-HD) treatment lead to RNase L activation? This would be a powerful experiment that strongly supports that the IBs prevent RNase L activation.

Response: Thank you for your insightful suggestion. This was indeed the outcome we considered as the ideal result. However, despite testing multiple experimental conditions, RNase L activation was not observed in RSV‑infected cells following 1,6‑HD treatment. We have included these findings, along with a detailed explanation, in the supplementary data and bellow attached image (lines 230-233, S8 Fig, revised manuscript). We hypothesize that this may result from the perturbative effects of 1,6‑HD, a phenomenon reported in several previous studies (DOI: 10.1016/j.jbc.2021.100260; DOI: 10.1016/j.jbc.2022.102365). We have discussed this phenomenon in the Discussion section (lines 317-321). This revision has further enhanced the logical and experimental consistency of our manuscript.

2. Fig. S7. RNA from mock-infected cells seemed to activate RNase L to some level. The RNA from RSV-infected cells did not lead to robust RNase L activation. It is unclear if dsRNA is host or viral, as RSV infection could lead to host dsRNA accumulation that could lead to RNase L activation. Does pre-expression of N block RNase L from RSV-infected cell derived RNA?

Response: Thank you for your valuable questions, which have helped us strengthen and clarify our manuscript.

1) In negative-sense single-stranded RNA viruses, copy-back defective viral genomes (cbDVGs) generated during genome replication are recognized as the primary source of dsRNA (DOI: 10.1371/journal.ppat.1007707). Notably, the dsRNA distribution pattern observed in Fig 3C (revised manuscript, zoom version was attached below) closely resembles the hollow droplet-like structures that co-localize with the N protein, consistent with previously characterized sites of RSV genome replication (DOI: 10.1038/s41467-017-00655-9). Collectively, these observations strongly suggest that the dsRNA detected in our study is RSV-derived (lines 294-300, revised manuscript).

2) To exclude the contribution of host-derived dsRNA, we confirmed that neither 1,6-HD treatment nor hypotonic shock induced detectable host dsRNA production (S7 Fig, revised manuscript). Furthermore, based on our current data, the minimal RNase L activation observed in cells transfected with total RNA extracted from mock-infected cells appears negligible (S7 Fig, revised manuscript). This interpretation is supported by the high ribosomal integrity number (RIN) value of 9.8, which is comparable to that of the mock-treated control group.
3) We believe that pre‑expression of the N protein alone is insufficient to block or sequester dsRNA from the OAS‑RNase L pathway. Formation of IBs requires at least both N and P proteins (DOI: 10.1128/mBio.01202-20). Furthermore, we assume that the sequestration of RSV dsRNA within IBs occurs in a replication‑dependent manner. This needs to be confirmed in future studies.

3. Briggs et al. 2025 (PMID: 40588419) showed that flavivirus RNA evades the effects of RNase L-mediated RNA decay, whereby RNase L can activate and degrade all cellular mRNA but viral RNA is not degraded. The authors should consider including this in their discussion on the role of viral replication complexes in evading the RNase L pathway.

Response: We thank you for your valuable advice. We have revised the Discussion section to include previously reported viral strategies for evading the RNase L pathway. (lines 276-282, revised manuscript)

Reviewer #3: This manuscript addresses how respiratory syncytial virus (RSV) inclusion bodies (IBs) formed by liquid–liquid phase separation (LLPS) enable immune evasion from the OAS–RNase L pathway. The authors show that RSV infection does not activate RNase L despite dsRNA production (Results, p.5–7, Fig 1), that ectopically activated RNase L fails to inhibit replication (Results, p.7–8, Fig 2), and that dsRNA is sequestered within IBs but becomes detectable after IB disruption with 1,6-hexanediol (Results, p.13–14, Fig 5). Purified dsRNA from infected cells activates the OAS–RNase L pathway (Results, p.15, Fig 6).
Strengths
• Logical experimental flow from lack of pathway activation → dsRNA sequestration → functional shielding.
• Convergent evidence from imaging and biochemical assays.
• Important conceptual contribution: IBs serve as both replication factories and immune-evasion compartments.
Weaknesses
• Reliance on 1,6-hexanediol as the sole LLPS perturbant. (lines 202-211, Fig 4B, Fig 5)
• No validation in IFN-competent primary cells. (lines 218-222, Fig 5)
• Lack of discussion of IBAGs, central to RSV RNA trafficking. (lines 56-60, 191, 294-300)
• No distinction between genomic RNA, replication intermediates, and viral mRNAs. (lines 64-66, 294-305)
• No acknowledgment of structural shielding mechanisms already shown by Gao et al., Structure, 2020/2021. (lines 300-305)
• Some overstatements of novelty (e.g., “IBs are viral factories,” which is long established).
Overall, the manuscript is carefully executed and of potential interest, but additional mechanistic validation and integration with known RNA biology are required before publication. (lines 60, 184, 198, 289)

Response: We sincerely appreciate the reviewer’s thoughtful evaluation and constructive feedback, which have greatly contributed to improving the rigor, clarity, and overall quality of our manuscript. In response, we have carefully revised the relevant sections in accordance with these suggestions and prepared a detailed, point‑by‑point reply outlining how each comment has been addressed in the revised version presented in “Part II.”.

**Part II – Major Issues: Key Experiments Required for Acceptance**
Please use this section to detail the key new experiments or modifications of existing experiments that should be absolutely required to validate study conclusions.

Generally, there should be no more than 3 such required experiments or major modifications for a "Major Revision" recommendation. If more than 3 experiments are necessary to validate the study conclusions, then you are encouraged to recommend "Reject".

Reviewer #1: The manuscript needs some minor clarifications.

I have clarified the issues raised in my mind in the Summary/Sp. Comments section. The authors may choose to move some supplemental figures to the results section and I would suggest the authors to decide.

Response: We thank you for your valuable suggestions. We have carefully reviewed your comments and provided corresponding responses in the Summary/Sp section. We would like to once again express our sincere appreciation for your thoughtful and constructive feedback. Your insightful advice has allowed us to address the shortcomings of our manuscript and develop a deeper, more comprehensive discussion.

Reviewer #2: The authors should provide a mechanism by which dsRNA sequestration in IBs prevent RNase L activation. For example, are OAS3 and RNase L excluded from RSV-IBs?

Response: We completely agree with your suggestion.

1) Condensate formation induced by OAS3 activation was not observed in RSV‑infected cells, whereas clear condensate formation was detected in cells transfected with poly (I:C). (lines 120-125, Fig 1F, revised manuscript)
2) In RSV-infected cells, OAS3 and RNase L were excluded from RSV-IBs. (line 195, S5 Fig, revised manuscript)
3) Furthermore, ectopically activated OAS3 formed condensates in RSV‑infected cells, these condensates did not overlap with RSV IBs. (lines 143-145, Fig 2C, revised manuscript)

Does 1,6-hexanediol (1,6-HD)-mediated dissolution of RSV-IBs lead to RNase L activation?

Response: Thank you for your insightful suggestion. This is already answered at “Part I – Summary_ Reviewer #2_ Minor comments No.1”. Briefly, Although RNase L activation would represent the expected outcome of successful dsRNA release, we did not observe RNase L activation in RSV-infected cells following 1,6-HD treatment despite testing multiple experimental conditions. These findings and a detailed interpretation have been included in S8A and B Fig and lines 230-233. We attribute this observation to the known off-target effects of 1,6-HD on RNase L activity, as documented in the literature (DOI: 10.1016/j.jbc.2021.100260; DOI: 10.1016/j.jbc.2022.102365). We believe that this revision has further enhanced the logical and experimental consistency of our study.

Reviewer #3: The key evidence for dsRNA shielding is derived from 1,6-hexanediol (1,6-HD) treatment (Results, p.13–14, Fig 5). However, 1,6-HD has broad off-target effects and may directly impair OAS–RNase L activity, confounding interpretation (Discussion, p.17). Prior work (Risso-Ballester et al., Nature, 2021) demonstrated that CPM-hardened IBs dissolve with 1,6-HD but resist hypotonic shock. I strongly recommend performing a 1,6-HD vs hypotonic shock ± CPM matrix, with readouts for IB material state (FRAP, morphology, p.11–12, Fig 4), dsRNA localization (IFA, p.11–13, Fig 4C), OAS–RNase L activation (rRNA cleavage, p.5–7, Fig 1; 2-5A; RNase L dimerization), and replication (Fig 1C, Fig 2B). Poly(I:C) transfection controls (Results, p.7–8, Fig 2A) should confirm pathway competence. This would firmly link IB material state to dsRNA shielding.

Response: We appreciate your constructive feedback and have carefully addressed your recommendations.

1) Disruption of LLPS via 1,6-hexanediol treatment and hypotonic shock
   dsRNA leakage from RSV-induced IBs into the cytosol was observed under both 1,6-HD treatment and hypotonic shock in CPM-untreated conditions (Fig 4, revised manuscript). The leaked dsRNA was localized to the periphery of IBs in both 1,6-HD treatment (1 min) and hypotonic shock (5 min) conditions (representative images provided below). Notably, following CPM treatment, only 1,6-HD induced detectable dsRNA leakage. In contrast, hypotonic shock failed to induce dsRNA release from CPM-hardened IBs (line 314, S10 Fig, revised manuscript). This observation is consistent with findings reported by Risso-Ballester et al. (DOI: 10.1038/s41586-021-03703-z). These results provide direct evidence that LLPS mechanism is essential for dsRNA sequestration within IBs. Supporting data and detailed interpretation have been incorporated into the revised manuscript (lines 203-222, Fig 4 and S10 Fig, revised manuscript).

2) The OAS–RNase L pathway was not activated following dsRNA release
   Notably, dsRNA released from RSV-induced IBs by 1,6-HD treatment or hypotonic shock did not activate OAS-RNase L-mediated rRNA cleavage (lines 230-233, S8 Fig, revised manuscript). We attribute this observation to the known off-target effects of 1,6-HD on RNase L activity, as documented in the literature (DOI: 10.1016/j.jbc.2021.100260; DOI: 10.1016/j.jbc.2022.102365). For hypotonic shock treatment, we hypothesize that while dsRNA leakage occurred, the incomplete release of viral dsRNA from IBs was insufficient to trigger OAS-RNase L-mediated rRNA cleavage. These interpretations and supporting data have been added to the revised manuscript (lines 317-330, S8 Fig, revised manuscript).
3) RSV replication kinetics following 1,6-HD treatment or hypotonic shock
   RSV replication was slightly increased under both 1,6-HD treatment and hypotonic shock compared with RSV infection alone (data presented below). We speculate that 1,6-HD and hypotonic shock exert broad effects on host cell physiology, including modulation of antiviral immune responses, thereby indirectly enhancing RSV replication. Although RSV dsRNA was released upon 1,6-HD treatment or hypotonic shock, the OAS–RNase L pathway was not activated, raising the possibility that OAS–RNase L is among the immune effector pathways that are broadly suppressed under these conditions.

2. Validation in an IFN-competent system.
All data are from A549 and HEp-2 cells (Methods, p.20–21), which have attenuated innate immune signaling. At least the key ± CPM, ± hypotonic shock dataset should be reproduced in primary human airway epithelial cells (NHBE or ALI cultures). Even a limited dataset here would provide crucial physiological relevance.

Response: We thank you for your valuable suggestions. We performed the same experiments described above using Primary Bronchial/Tracheal Epithelial cells (ATCC, PCS-300-010) and obtained consistent results. These findings have been incorporated into the main manuscript along with a corresponding explanation (lines 218-222, Fig 5, S7B Fig, S8B Fig, S10B Fig, revised manuscript). This addition extends our observations from standard cell lines to clinically relevant primary cells, thereby strengthening the translational relevance of our study.

3. Incomplete treatment of RNA biology and structural mechanisms.

The manuscript convincingly shows dsRNA shielding but omits critical aspects of RSV RNA biology:

• IBAGs omitted: Inclusion body–associated granules (IBAGs) are the recognized substructures where viral mRNAs accumulate and exit IBs (Rincheval et al., Nat Commun 2017). Their absence from the Introduction, Results (p.11–12, Fig 4), and Discussion (p.16–18) is striking.

Response: This is a very important point, and we apologize for omitting discussion of this critical aspect. We have revised the introduction (lines 56-60, revised manuscript), results (line 191, Fig 3C, revised manuscript), and discussion (lines 294–300, revised manuscript) to highlight the substructure known as IBAGs.

The IBAGs substructure and M2-1–mediated RNA trafficking represent critical determinants for inferring the origin and species of the dsRNA detected in our study. Briefly, the dsRNA pattern detected in Fig 3C (revised manuscript, zoom version was attached below) was localized within IBs but excluded from the IBAG substructure. In negative-sense single-stranded RNA viruses, the primary source of dsRNA is copy-back defective viral genomes (cbDVGs) generated during the genome replication process (10.1371/journal.ppat.1007707). Genome replication occurs within the IBs matrix but outside IBAGs. Furthermore, M2-1, which traffics newly synthesized viral RNA to IBAGs, exhibits a lower binding affinity for dsRNA compared to single-stranded RNA, potentially accounting for the exclusion of dsRNA from these substructures. Accordingly, the observed localization of dsRNA within IBs yet excluded from IBAGs is consistent with this model (Fig 3C, revised manuscript).

• RNA species not distinguished: The dsRNA detected (Results, p.9–11, Fig 3C) could represent genomic RNA, replication intermediates, or mRNA duplexes. The authors should at least discuss this limitation, and ideally provide RNA FISH or qPCR data for viral mRNAs.

Response: This suggestion has precisely identified a critical limitation of our study. We sincerely appreciate the reviewer’s careful and thoughtful consideration. We have made every effort to clarify this aspect based on our current data and understanding.

In negative-sense single-stranded RNA viruses, the primary source of dsRNA is known to be copy-back defective viral genomes (cbDVGs) generated during genome replication (DOI: 10.1371/journal.ppat.1007707). In Fig. 3C (revised manuscript), dsRNA was detected as hollow droplet-like structures that co-localize with N protein, consistent with previously defined RSV genome replication sites located outside IBAGs. Collectively, these observations strongly suggest that the dsRNA detected in our study represents viral replication intermediates, likely cbDVGs. However, we acknowledge that the presence of other dsRNA species cannot be completely excluded based on our current analysis. We plan to perform detailed characterization of dsRNA species and investigate the mechanisms underlying dsRNA synthesis during RSV replication in future studies. We have incorporated interpretation into the revised manuscript (lines 64-66, 294-300).

• Structural shielding not discussed: Gao et al., Structure, 2020/2021 demonstrated that RSV gene-end ds mRNA is directly occluded by M2-1/P–RNA complexes. This structural shielding mechanism should be acknowledged and integrated into the Discussion (p.16–18).
Without addressing these points, the mechanistic framework risks being oversimplified as LLPS-only shielding.

Response: We thank you for your valuable suggestions.
The crystal structure reported by Gao et al. shows a partial duplex formed between two 7-nt SH gene-end RNAs. Critically, M2-1 interacts exclusively with the non-paired, single-stranded regions of these RNAs. The duplex region itself has no contact with M2-1. Gao et al. explicitly note that M2-1 exhibits "low affinity for double-strand RNA" and suggest the base-pairing "might be due to crystallography packing" (Gao et al., 2020, Results section, DOI: 10.1016/j.str.2020.07.001), indicating this is likely a crystallographic artifact rather than a physiologically relevant RNA structure. These interpretations have been added to the revised manuscript (lines 300-305, revised manuscript).

**Part III – Minor Issues: Editorial and Data Presentation Modifications**
Please use this section for editorial suggestions as well as relatively minor modifications of existing data that would enhance clarity.

Reviewer #1: The authors describes how cleverly RSV manipulates innate immune system of the host to attenuate a prompt and robust antiviral immunity. The manuscript describes a nice piece of intelligent work.

Reviewer #2: None

Reviewer #3: (No Response)
