# Supplementary material for: Correction: Liquid-liquid phase separation mediated immune evasion of respiratory syncytial virus against oligoadenylate synthetase-RNase L pathway
Source: PLoS Pathog. 2026 May 5;22(5):e1014193. doi: 10.1371/journal.ppat.1014193 (PMC13143112; doi:10.1371/journal.ppat.1014193)
Supplement: S5 Fig — (A) A549-RNase L KO stably expressing GFP-RNase L and mRuby2-OAS3 cells were infected with RSV A2 at an MOI of 2, stained with anti-N antibody (green) at 24 h post-infection, and imaged using an LSM 980 confocal microscope. (B) The white line indicated the rack of a line intensity profile. Scale bar, 10 μm. (DOCX) [file ppat.1014193.s001.docx]

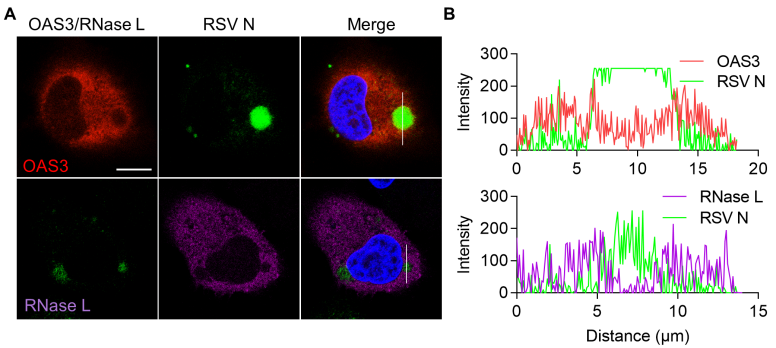


**S5 Fig. OAS3 and RNase L were excluded from inclusion bodies (IBs).** (A) A549-RNase L KO stably expressing GFP-RNase L and mRuby2-OAS3 cells were infected with RSV A2 at an MOI of 2, stained with anti-N antibody (green) at 24 h post-infection, and imaged using an LSM 980 confocal microscope. (B) The white line indicated the rack of a line intensity profile. Scale bar, 10 μm.
